# Protocol for a cluster randomised controlled trial of secondary distribution of hepatitis C self-testing within the context of a house-to-house hepatitis C micro-elimination programme in Karachi, Pakistan

**DOI:** 10.1186/s12889-022-13125-9

**Published:** 2022-04-09

**Authors:** Sonjelle Shilton, Dania Ali, Alyia Hasnain, Adeel Abid, Jessica Markby, Muhammad S. Jamil, Niklas Luhmann, Pamela Nabeta, Stefano Ongarello, Elena Ivanova Reipold, Saeed Hamid

**Affiliations:** 1 FIND, Campus Biotech, Chemin des Mines 9, 1202 Geneva, Switzerland; 2 Aga Khan University, Stadium Road, Karachi, Pakistan; 3 WHO Global HIV, Hepatitis & STI programmes, Avenue Appia 20, 1202 Geneva, Switzerland

**Keywords:** Hepatitis C, Self-testing, Micro-elimination, Pakistan, Testing uptake

## Abstract

**Background:**

Globally, just 21% of the estimated 58 million people living with hepatitis C virus (HCV) know their status. Thus, there is considerable need to scale-up HCV testing if the World Health Organization (WHO) 2030 hepatitis elimination goals are to be achieved. HCV self-testing may assist with this; however, there are currently no data on the real-world impact of HCV self-testing.

With an estimated 5% of the general population living with HCV, Pakistan has the second highest HCV burden in the world. This study aims to evaluate the acceptability and impact of home delivery of HCV self-testing for secondary distribution in the context of a house-to-house HCV micro-elimination programme in Pakistan.

**Methods:**

This is a parallel group, non-blinded, cluster randomised trial comparing secondary distribution of HCV self-testing with secondary distribution of information pamphlets encouraging individuals to visit a testing facility for HCV screening. The cluster allocation ratio is 1:1. Clusters will be randomised either to HCV self-testing distributed via study staff or control clusters where information on HCV will be given and the participant will be requested to attend their local hospital for HCV screening. In both clusters, only households with a member who has not yet been screened as part of the larger micro-elimination project will be included. The primary outcome is the number and proportion of participants who report completion of testing. Secondary outcomes include the number and proportion of participants who a) receive a positive result and are made aware of their status, b) are referred to and complete HCV RNA confirmatory testing, and c) start treatment. Acceptability, feasibility, attitudes towards HCV testing, and cost will also be evaluated. The target sample size is 2,000 participants.

**Discussion:**

This study will provide the first ever evidence regarding secondary distribution of HCV self-testing. By comparing HCV self-testing with facility-based testing, we will assess whether HCV self-testing increases the uptake of HCV testing. The findings will inform micro-elimination programmes and determine whether HCV self-testing can enable individuals to be reached who may otherwise be missed.

**Trial Registration:**

This study and was registered on clinicaltrials.gov (NCT04971538) 21 July 2021.

**Supplementary Information:**

The online version contains supplementary material available at 10.1186/s12889-022-13125-9.

## Background

Hepatitis C virus (HCV) is a leading cause of liver cancer globally; however, just 21% of the 58 million individuals living with HCV have been diagnosed [1]. The diagnosis of HCV currently involves a two-step process: first, a person receives a facility-based serological test for HCV antibodies, then, if the result is positive, they receive a second test to assess for viraemia [2]. The World Health Organization (WHO) recommends treating all individuals with viraemia with directing-acting antiviral (DAA) therapy [2].

There is an urgent need to scale-up HCV testing to achieve the WHO 2030 hepatitis elimination goals [3]. In July 2021, WHO announced the first ever recommendations for HCV self-testing, which state that HCV self-testing should be offered as an additional approach to serological testing for HCV antibodies [4]. Self-testing refers to a process where an individual, either alone or with someone they trust, performs a test and interprets the result [4].

HCV self-testing has been shown to be a feasible and acceptable testing approach that individuals perceive to be a tool that offers a convenient and private way for them to learn of their hepatitis C status [5–10]. However, there are currently no data on the real-world impact of HCV self-testing, including its effect on uptake of testing and linkage to care.

With an estimated 5% of its general population living with HCV, Pakistan has the second highest burden of this infection in the world [11]. A major scale-up of testing and treatment is needed if Pakistan is to eliminate HCV [12]. The current micro-elimination programme, led by Aga Khan University (AKU), is taking place in three Union Councils (UC) of Malir District in Karachi, Pakistan: UC-9 (Darsano Chano), UC-10 (Malh) and UC-11 (Murad Memon). This micro-elimination programme brings HCV screening, diagnostic and treatment services to people’s doorsteps and aimed to test around 30,000 people, as described previously [13]. However, the current programme has not been able to screen the entirety of the planned coverage area due to an estimated 39% of household members being absent when AKU staff made their field visits. The present study will assess whether secondary distribution of HCV self-testing can increase testing uptake among individuals who were unavailable during the visit by testing campaign staff. In the context of HIV, the secondary distribution of HIV self-tests during door-to-door testing campaigns substantially increased testing coverage (by 21%) among absent individuals or those who declined to participate [14]. The current study will be the first to explore this mode of delivery of self-testing for HCV.

### Study aim and objectives

This study aims to evaluate the acceptability and impact of the secondary distribution of HCV self-tests in the context of a house-to-house HCV micro-elimination programme in Malir district, Karachi division, Pakistan. The primary objective is to assess the impact of HCV self-testing on HCV antibody testing rates. The secondary objectives are to assess the impact of HCV self-testing on linkage to and completion of HCV RNA confirmatory testing in HCV antibody-positive individuals and treatment initiation in HCV RNA-positive individuals eligible to begin treatment; to assess the acceptability and feasibility of HCV self-testing; and to assess the cost and cost-effectiveness of HCV self-testing compared with facility-based testing.

## Methods/design

### Design

This is a parallel group, non-blinded, cluster randomised superiority trial comparing the secondary distribution of HCV self-tests with the secondary distribution of information pamphlets encouraging individuals to visit a testing facility for HCV screening. A subset of participants (*n* = 120) from both the intervention and control groups will be asked to participate in face-to-face interviews regarding their perceptions of the testing experience, to provide insights into the acceptability and feasibility of HCV self-testing. The study timeline is shown in Table 1.

**Table 1:** Study timeline

	**Enrolment**	**Follow-up for all participants**	**Follow-up for HCV antibody-positive participants**
**Procedure**	**Day 1**	**Months 1–2**	**Months 1–8**
Inclusion and exclusion criteria	X		
Informed consent obtained	X		
HCV antibody testing (either HCV self-test left with the household or participants in the control group attend Memon Goth Hospital)	X	X	
Collection of test results and survey of perceptions of the testing process		X	
Referral of HCV antibody-positive individuals for confirmatory testing and linkage to care		X	X
Linking of data for the RDT (control group) and RNA test and treatment initiation (as applicable for the intervention and control groups)		X	X
Adverse event (AE)/serious adverse event (SAE) review	X	X	X
Cost data collection and analysis	X	X	X

### Study setting

The study will take place in the Malir district of Karachi, Pakistan. Darsano Chano (UC9) is in a rural area and has a population of approximately 10,000 individuals. Malh (UC10) is peri-urban and has a total population of approximately 50,000 individuals. Both UCs have small, well-established community clinics, which have formed the hub of the larger ongoing HCV micro-elimination programme. UC9 and UC10 have approximately 2,000 and 10,000 households, respectively, with an average of five family members per household.

**Fig. 1:**
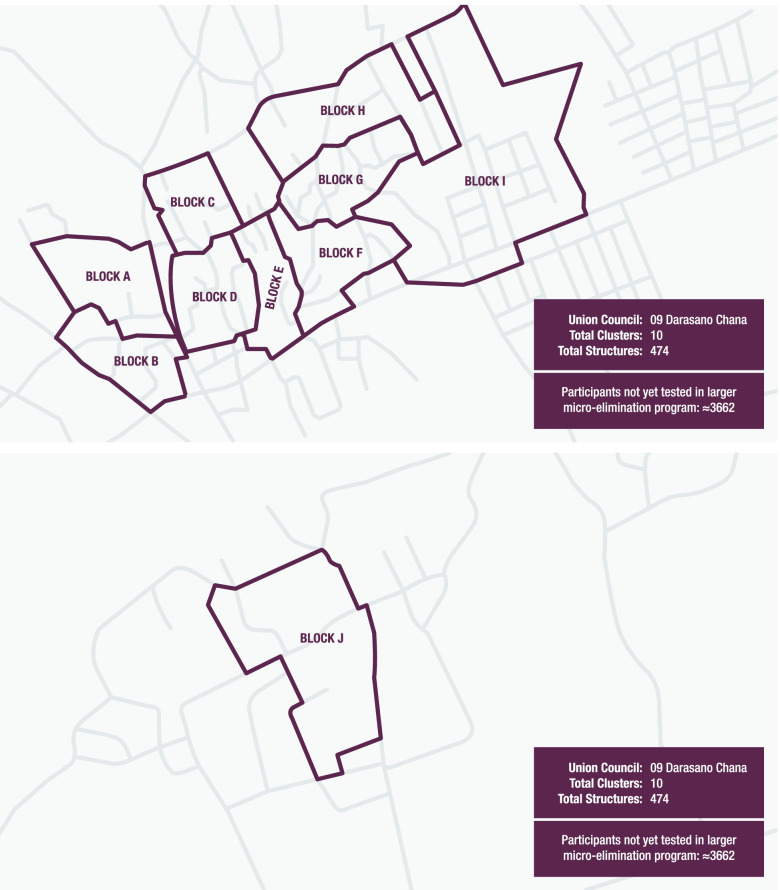
Map of UC9. Maps created using Adobe Illustrator 25.4 for Windows. https://www.adobe.com/ro/products/illustrator.html?skwcid=AL!3085!3!341240180390!e!!g!!adobe%20illustrator&mv=search&sdid=KCJMVLF6&ef_id=CjwKCAiAgbiQBhAHEiwAuQ6Bknh2k612ZgbuSDwblwUKE5L1T1KaZATGaxTwAGIkknQrdcWhGZ6JrxoCtNAQAvD_BwE:G:s&s_kwcid=AL!3085!3!341240180390!e!!g!!adobe%20illustrator!1478481991!58339249918&gclid=CjwKCAiAgbiQBhAHEiwAuQ6Bknh2k612ZgbuSDwblwUKE5L1T1KaZATGaxTwAGIkknQrdcWhGZ6JrxoCtNAQAvD_BwE

**Fig. 2:**
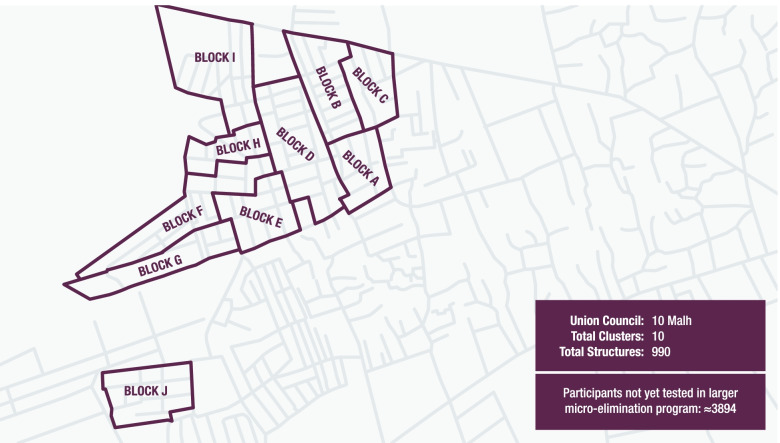
Map of UC10. Maps created using Adobe Illustrator 25.4 for Windows. https://www.adobe.com/ro/products/illustrator.html?skwcid=AL!3085!3!341240180390!e!!g!!adobe%20illustrator&mv=search&sdid=KCJMVLF6&ef_id=CjwKCAiAgbiQBhAHEiwAuQ6Bknh2k612ZgbuSDwblwUKE5L1T1KaZATGaxTwAGIkknQrdcWhGZ6JrxoCtNAQAvD_BwE:G:s&s_kwcid=AL!3085!3!341240180390!e!!g!!adobe%20illustrator!1478481991!58339249918&gclid=CjwKCAiAgbiQBhAHEiwAuQ6Bknh2k612ZgbuSDwblwUKE5L1T1KaZATGaxTwAGIkknQrdcWhGZ6JrxoCtNAQAvD_BwE

The study will take place at the homes of eligible adults visited by study staff. Based on the current micro-elimination programme, an estimated 7,500 participants remain in both UC9 and UC10 who have not yet been tested as part of the larger micro-elimination programme. Each UC has been subdivided into ten blocks/clusters, based on neighbourhood geography, as shown in Figs. 1 and 2; these clusters have not yet been randomised, but will be matched-pair randomised on a 1:1 basis.

Participants in the control clusters will be directed to Memon Goth Hospital for facility-based HCV testing; if a participant tests positive by HCV rapid diagnostic test (RDT), they will immediately have blood drawn for viraemia testing. As described below, for those in the self-test group who report a positive self-test, a member of the AKU team will come to their house to perform a blood draw for viraemia testing and liver staging. Treatment for both groups will be provided at each participant’s doorstep, free of charge, through a partnership between AKU and The Liver Foundation.

As there is currently no quality-assured HCV self-test available, the professional-use OraQuick® HCV Rapid Antibody Test, which has been adapted by the manufacturer for self-testing by being repackaged and labelled with instructions for use, will be used in this trial. The OraQuick® HCV Rapid Antibody Test is prequalified by WHO and CE-marked for professional use (sensitivity 98.1%, specificity 99.6%). The OraQuick® HCV Self-Test kits provided to participants in the intervention group will be labelled for Research Use Only (RUO).

### Eligibility criteria

Participants are only eligible to be included in the trial if they meet all of the following inclusion criteria:

-  ≥ 18 years of age
- Residing in UC9 or 10
- Not yet reached for testing as part of the larger micro-elimination programme
- Not known to be HCV antibody-positive
- Not tested for HCV within the past 6 months
- Eligible for inclusion in the larger micro-elimination programme

Participants will be excluded from the trial if they are at home on the day that AKU study staff visit to either leave an HCV self-test or the information materials encouraging those who were previously missed to attend for facility-based HCV testing.

### Informed consent

Study staff will explain the nature of the study to the most senior member of the household present, and who is aged 18 years or more, in a language understandable to her/him, and answer any questions they have about the study. The study staff will leave an informed consent form along with the other study materials. During the telephone call to report results, the participant will be verbally asked if they consent to participate in this study. The 120 people who will be surveyed in the face-to-face interviews will also be given the opportunity to provide informed consent.

### Trial processes and intervention

The neighbourhoods in UCs 9 and 10 will be grouped into clusters. These clusters will be matched in terms of similarity of geography (peri-urban or rural), population size, and age demographics. Each member of a matched cluster pair will be randomised to the intervention or control groups.

**Fig. 3:**
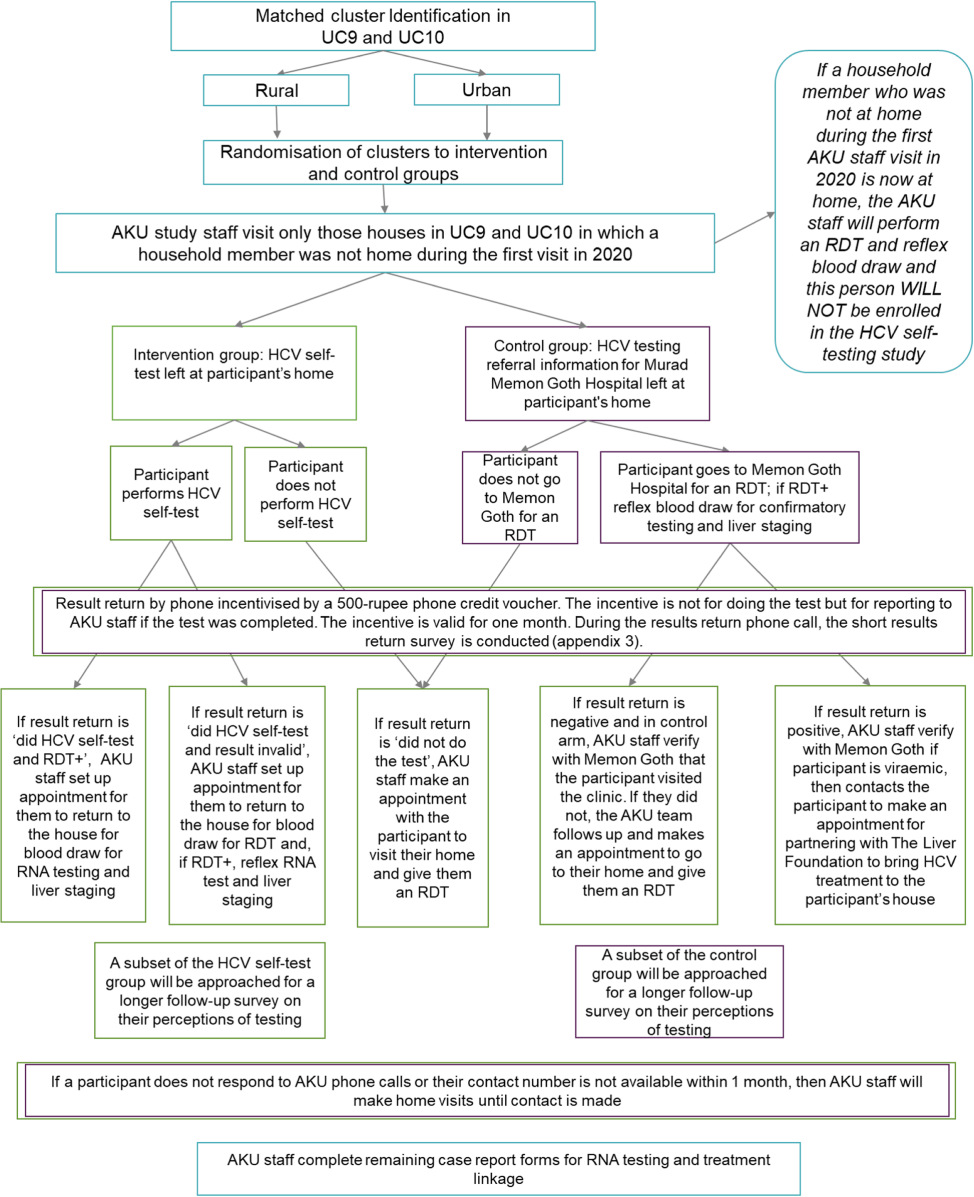
Trial flow diagram

As outlined in Fig. 3, AKU study staff will return to the households where individuals were missed during the first round of house-to-house screening carried out as part of the larger micro-elimination programme. In the intervention group, the study staff will leave as many HCV self-tests as there were individuals missed from the first round of house-to-house screening who are still not present during the visit, as well as instructions for use, with the most senior member of the household present. The study team will also explain the HCV self-testing process, using a standardised explanatory checklist, to that same senior member of the household; they will also leave a mobile phone number for the participant to call if they need help in conducting the test. Participants will be provided with various types of support to minimise the occurrence of errors during the self-testing process and any possible confusion in interpretation of the test results. Printed instructions for use in Urdu will be delivered with the test kits, which will also contain pictorial guides on how to use the test. Participants will have access to a live chat facility and a call centre.

In the control group, if the participant(s) are not at home, the study staff will leave information about HCV testing and direct them to the nearest clinic that offers HCV testing services; the participant(s) will not be left an HCV self-test. Study staff will however leave a mobile phone number for the participant(s) to call if they need further information on HCV testing.

In both groups, if no-one is at home or no one aged 18 years or more is present, the study staff will make as many return visits as necessary to contact a household member.

A small incentive, comprising a 500 rupee (approximately 3 USD) phone credit voucher, will be provided to participants in both the intervention and control groups for any information they return via a phone call, including whether they took the test and, if so, what the result was. During this return phone call, the study team will follow up with the participant to inquire whether they completed the test, record any test results, and conduct a brief interview.

For all participants who report a positive HCV antibody test, study staff will make an appointment to return to their house to enrol them in the larger micro-elimination programme. During this home visit, they will complete a blood draw for HCV viraemia testing and APRI (aspartate aminotransferase to platelet ratio index) liver staging, to be performed at Memon Goth Hospital. The viraemia testing will be performed using an HCV core antigen assay (ARCHITECT HCV Ag assay, Abbott® Diagnostics). If a participant’s result is in the grey zone (≥ 3 to ≤ 10 fmol), a second sample from the same participant will be tested for HCV RNA (Xpert HCV Viral Load, GeneXpert® IV, Cepheid, France) to determine viraemia. If the participant is viraemic, further care and treatment will be provided to their doorstep. All participants who report a negative HCV antibody test will be encouraged to seek a retest, as per the national guidance.

Approximately 1 month following the start of enrolment, face-to-face interviews will be conducted with a subset of participants in both the intervention and control groups, to explore their perceptions of the testing process.

As this study is intended to measure whether there is an increase in testing following the secondary distribution of HCV self-testing compared with the standard of care facility-based testing, facility-based testing was chosen as the comparator. In both groups, if a previously missed individual is at home when a study staff member visits, they will enrol the person in the larger micro-elimination programme (and not this study).

### Strategies to increase recruitment

As part of the larger micro-elimination programme, AKU has already established a community oversight board comprising community leaders from neighbourhoods UC 9 and 10. This community oversight board has been informed about this new study component. AKU works with the community oversight board to sensitise each neighbourhood to the study, to facilitate information sharing and encourage interest in participating in the study.

### Outcomes

The primary outcome is to assess whether the proportion of participants who report completing HCV antibody testing in the intervention group is greater than that of the participants in the control group by a margin of 20%.

The secondary outcome measures are as follows.

- The number and estimate of the proportion of HCV antibody-positive individuals made aware of their antibody status in the intervention versus the control group
- The number and estimate of the proportion of HCV antibody-positive individuals who are referred for and complete HCV RNA confirmatory testing in the intervention versus the control group
- The number and estimate of the proportion of HCV RNA-positive individuals who begin treatment in the intervention versus the control group
- The cost per test completed and the cost per person diagnosed (serology, RNA) in the intervention versus the control group
- An analysis of the knowledge, attitudes and practice survey responses will be conducted, using proportions and means

### Participant timeline

#### Sample size

A minimum of 2,000 participants who meet the inclusion criteria will be enrolled in the study. There will be up to 20 clusters, each comprising up to 150 to 200 households: 5 intervention clusters each in rural and peri-urban areas and 5 control clusters each in rural and peri-urban areas. The study is powered to detect at least a 20% between-group difference in HCV antibody self-testing, based on 80% statistical power and an alpha level of 0.05.

The survey of participants’ perceptions in relation to testing will include face-to-face interviews with 60 participants from each of the intervention and control groups. This is guided by empirical thinking, considering the feasibility with regards to the time and staff required to conduct the in-person interviews. The aim of these interviews is to further expand our understanding of preferences around HCV self-testing; there is no intention to set specific targets for measurement.

For the primary objective, the null hypothesis is that there is no significant difference in the proportion of individuals reporting HCV test results in the intervention versus control groups. The alternative hypothesis is that home delivery of HCV self-tests will significantly increase the proportion of individuals reporting HCV test results.

The study is not powered to detect significant differences in the secondary objectives. However, similar to the primary objective, the null hypothesis would be no significant difference between the intervention versus control groups. The alternative hypothesis would that home delivery of HCV self-tests will result in significantly higher numbers of HCV-infected individuals being diagnosed, linked to care and treated, making it a cost-effective approach to HCV case finding and elimination.

#### Assignment of interventions: allocation

Each member (neighbourhood block) of a matched cluster pair will be randomly allocated to the intervention or control group using a simple, computer-generated randomisation list. Each matched pair will be established based on similarities in type of geography (urban/peri-urban or rural), population size, sex distribution, age range, income and literacy. Within each UC and type of cluster (rural versus peri-urban), clusters will be put into pairs based on average household size (i.e. total estimated population in the cluster divided by the number of structures in the cluster). For example, the two rural clusters with the two largest average household sizes will be paired, followed by the next two rural clusters with the third and fourth largest average household sizes, and so on. Similar pairing will be performed for peri-urban clusters. If there are more than two clusters with the same average household size, then the estimated average age will be used as a tiebreaker, and pairs will be selected based on the closest estimated average age in those clusters. Once all clusters have been put into pairs, each member of a pair will be randomised to the intervention or control group.

For the face-to-face interviews, a subset of the main participants will be selected. Based on the study cluster assignment and testing results, study participants will be divided into the following eight subgroups:

Intervention group:

- In a rural cluster in the intervention group and completed a test
- In a rural cluster in the intervention group, but did not complete a test
- In a peri-urban cluster in the intervention group and completed a test
- In a peri-urban cluster in the intervention group, but did not complete a test

Control group:

- In a rural cluster in the control group and completed a test
- In a rural cluster in the control group, but did not complete a test
- In a peri-urban cluster in the control group and completed a test
- In a peri-urban cluster in the control group, but did not complete a test

For each subgroup, a randomly ordered list of participants will be generated via computerised randomisation. Then, for each subgroup, participants will be contacted by study staff in descending order on the list until a maximum of 15 individuals in each subgroup have completed the survey.

The trial is not blinded, and the participants and study staff know which group the participants are assigned to; therefore, no additional concealment mechanisms will be used.

### Data collection and management

All participant data relating to the study will be recorded in source documents and transcribed onto a paper case report form by the study team. The data will then be entered into OpenClinica Enterprise Edition, version 4.0, which is secure and password-protected.

Data for the results return interview (supplementary material, annex 1) will be collected over the phone. Data collection for the follow-up interviews among a subset of participants (supplementary material, annex 2) will be conducted face-to-face. Both the results return interview and the follow-up interview tools were created specifically for this study. Data relating to further HCV RNA testing and linkage to care will be extracted from records at Memon Goth Hospital and AKU treatment partners.

Costing information with regards to costs per test and costs per person diagnosed with HCV will be collected and analysed. The data collected will be used to conduct economic analyses that will include the following:

- The cost of delivering the HCV self-tests, including associated human resources time
- The cost of conducting an RDT at a local clinic
- The number and type of diagnostic and therapeutic tests and procedures
- The number of healthcare facility visits before a diagnosis is made, or time to results

#### Confidentiality

Participants will be assigned a unique identifier, generated by FIND. Any participant records or datasets that are transferred to FIND will include this identifier only; no participant names or any other information that would make the participant identifiable will be transferred.

### Statistical methods

The data will be analysed using R version 4.1 or higher. A summary of statistical analyses that will be completed by the endpoint is shown in Table 2. The primary analysis will be performed using an intention-to-test analysis. The incentivisation methods described above will be employed to minimise missing data. The complete analysis plan is described in the study statistical analysis plan, which was drafted at the time the protocol was developed.

**Table 2:** Statistical analyses to be completed by the endpoint

**Endpoint**	**Statistical analysis method**
1.1 The number and estimate of the proportion of participants who report completing HCV antibody testing in the intervention group 1.2 To assess whether the proportion of participants who report completing HCV antibody testing in the intervention group is greater than that of participants in the control group, by a margin of 20%	Test of proportions, comparing the proportion of individuals reporting HCV test results in the intervention versus control groups and between intervention groups
2.1 The number and estimate of the proportion of HCV antibody-positive individuals made aware of their status in the intervention versus the control group	Descriptive statistics; statistical tests comparing the number of individuals in the intervention versus the control group and between intervention groups
2.2 The number and estimate of the proportion of HCV antibody-positive individuals who are referred to and complete HCV RNA confirmatory testing in the intervention versus the control group	
2.3 The number and estimate of the proportion of HCV RNA-positive individuals who begin treatment in the intervention versus the control group	
2.4 Analysis of survey responses using proportions and means	Descriptive statistics for survey responses (e.g. proportions, means)
2.5 Cost per test completed and cost per person diagnosed (serology, RNA) in the intervention versus the control group	Ingredients-based cost calculation approach

The estimates of the study outcomes are proportions pfo,X, (fo = favourable outcome, X = element of {intervention, control}). They will be based on the definitions shown in Table 3, for estimation as well as for between-arm comparison.

**Table 3:** Analysis table for primary and secondary analyses

Outcome							
		Favourable; test completed				Non-favourable; test not completed	Total
Test result and consequence 1		Positive and referred to further testing	Positive and not referred to further testing	negative	No result		
Test result and consequence 2		Positive and treated	Positive and not treated				
Study arm	Intervention	a	b	c	d	e	(a + b + c + d + e)
	Control	f	g	h	i	j	(f + g + h + i + j)
Total		(a + b + c + e + f + g + h + i)				(e + j)	total

The consequences (referred, treated) will be analysed independently of each other

The number and estimate of the proportion of participants who report completing HCV antibody testing in the intervention group will be calculated as:

$$\mathrm{pfo},\mathrm{I }= (\mathrm{a}+\mathrm{b}+\mathrm{c}+\mathrm{d})/(\mathrm{a}+\mathrm{b}+\mathrm{c}+\mathrm{d}+\mathrm{e})$$

$$\mathrm{pfo},\mathrm{C }= (\mathrm{f}+\mathrm{g}+\mathrm{h}+\mathrm{i})/(\mathrm{f}+\mathrm{g}+\mathrm{h}+\mathrm{i}+\mathrm{j})$$

The absolute and relative proportions will be calculated separately by arm (all arms) and reported with their two-sided 95% Wilson score confidence interval.

The difference pfo,I—pfo,C will be assessed using a one-sided test with a margin of 20%, by applying the following hypothesis:

$$\mathrm{H}0:\mathrm{ pfo},\mathrm{I }-\mathrm{ pfo},\mathrm{C }\le 20\mathrm{\%}$$

$$\mathrm{HA}:\mathrm{ pfo},\mathrm{I }-\mathrm{ pfo},\mathrm{C }> 20\mathrm{\%}$$

The difference of the proportions in the intervention and control arms will be reported, together with its confidence interval (see below) and the *p*-value related to the null hypothesis mentioned above.

The alpha level for the analysis will be set at 0.025 (1 one-sided test with an alpha level of 0.025).

The difference and its confidence interval will be estimated using a generalised binary mixed logit model with “cluster pair” as a random effect and “group” as a fixed effect (e.g. using the R function “lmer”). The two-sided 95% asymptotic confidence interval will be used to derive the one-sided lower 97.5% confidence limit, which will be used for interpretation. In addition, the odds ratio and its confidence interval will be reported.

#### Oversight and monitoring

The support for this study is provided by:

- The principal investigator, who has overall responsibility for the supervision of the study and medical responsibility for the participants
- An AKU study coordinator, who will ensure that the field visits to the clusters and participants take place according to the schedule and that study procedures are followed
- AKU study team members, who will conduct the field visits, record study data, organise payment of incentives to participants who complete the surveys, and ensure linkage to the larger micro-elimination programme as necessary

The study team meets on a weekly basis. There is no data monitoring committee for this study. This decision was based on the lack of serious adverse events in previous feasibility and acceptability studies of HCV self-testing completed in Pakistan and six other countries [4], as well the fact that many large-scale HIV self-testing studies and pilots have been conducted without such committees.

The occurrence of any social harms will be monitored by the community leaders’ steering committee. This group will follow-up to address any issues that arise and provide support where necessary. A telephone hotline will also be made available to participants. Any reports of social harms will be aggregated into monthly reports.

This study will be guided by a risk-based monitoring approach. FIND will engage an independent trial monitor, who will conduct a source document review and data checks on a random selection of 10% of the case report forms. Any protocol amendments will be notified to the AKU Ethics Review Committee.

### Dissemination plans

The results of this research will be widely disseminated, targeting community groups, academia, implementers and policymakers, using a variety of methods. A national dissemination event will be held, which will aim to bring together community and health professionals. The results of the study will be written up and submitted for presentation at international conferences and publication in peer-reviewed journals.

## Discussion

Globally, almost four out of five individuals with chronic HCV infection were estimated to be undiagnosed in 2019 [1]. The current facility- and community-based testing approaches alone are not reaching a major proportion of those who remain undiagnosed for HCV. New approaches are needed to address this gap. WHO now recommends HCV self-testing as an additional approach to HCV testing services [3]. We are conducting the first study of real-world implementation of HCV self-testing, in a pragmatic, cluster randomised trial in a setting where there is a high burden of HCV, to assess the uptake of testing and related outcomes.

### Strengths

This study will provide the first ever evidence use of secondary distribution of HCV self-testing. By using a randomised approach to compare HCV self-testing with facility-based testing, we will gain insights into whether the availability of HCV self-testing increases the uptake of HCV testing. In the context of the COVID-19 pandemic, wherein individuals’ movements around Pakistan may become very restricted, the provision of home-based HCV self-testing may help to mitigate some of the negative impacts the pandemic has had on the hepatitis programme.

### Limitations

This study has some important limitations that will be considered and described in the study report. The uptake of testing in the control arm may be affected by the geographical location of the participant and the distance to Memon Goth Hospital, as well as by the ongoing COVID-19 pandemic, which may affect participants’ willingness to visit a healthcare facility. The interview questionnaires have a multiple-choice design, which may not capture some important context-specific aspects.

Cluster randomised trials have the possibility of cross-contamination bias; however, in this study the risk of cross-contamination bias is limited, because the control group will not have access to HCV self-tests as they are not currently on the market in Pakistan. Given that the time between enrolment and result return is expected to take up to a maximum of 1 month, incentives are offered for the return of results, and “did not complete the test” is an option for the return of the results; therefore we are not anticipating a large attrition bias. Detection bias will be minimised by clearly defining the study procedures used to measure the outcomes and by using the same procedures and data collection tools in both the intervention and control groups, for example the result reporting procedure is the same across both the intervention and control groups, with the same data collection tool, result reporting procedure, and result reporting timeframe.

The WHO HCV self-testing guidelines recommend that HCV self-testing delivery models, support tools and referral pathways are designed and adapted according to the local context and community preferences. This study will provide an opportunity to pilot implementation approaches and explore their feasibility in the context of micro-elimination efforts. These approaches can be refined and optimised for broader use in national testing plans, based on the lessons learned. Given the significant burden of HCV in Pakistan and elsewhere, this study will inform efforts to scale-up approaches to diagnose individuals with HCV who would otherwise not access testing and link them to effective treatment. Further studies can be conducted to assess other implementation models in different settings.

## Trial status

Protocol V 1.0, 6 July 2021. Enrolment began in November 2021 and is expected to be completed in Q1 of 2022.

## Supplementary Information

**Additional file 1. **

**Additional file 2. **

## Data Availability

Not applicable. The final dataset will be housed by FIND and will be available upon reasonable request to the corresponding author, in accordance with the data sharing policies of FIND and AKU.

## Abbreviations

AKU: Aga Kha University
DAA: Direct-acting antiviral
FIND: Foundation for Innovative New Diagnostics
HCV: Hepatitis C virus
HIV: Human immunodeficiency virus
RDT: Rapid diagnostic test
RNA: Ribonucleic acid
UC: Union council
WHO: World Health Organization
