# Supplementary material for: Protocol for a cluster randomised controlled trial of secondary distribution of hepatitis C self-testing within the context of a house-to-house hepatitis C micro-elimination programme in Karachi, Pakistan
Source: BMC Public Health. 2022 Apr 9;22:696. doi: 10.1186/s12889-022-13125-9 (PMC8994067; doi:10.1186/s12889-022-13125-9)
Supplement: Supplementary file 1 — Additional file 1. [file 12889_2022_13125_MOESM1_ESM.docx]

## Results return brief phone interview

**HEPATITIS C SELF-TESTING STUDY**

**RESULTS RETURN PARTICIPANT SURVEY**

**STUDY ID:**

**SURVEY DATE:** / ___/ ___/ __/ (day/month/year)

***Conducted at time of follow up to see if participant used self test or went to local site to get tested. Participant is eligible for 500-rupee phone credit voucher for completing the survey no matter the answer to if they completed the test**

**INFORMATION TO PARTICIPANTS**

**This questionnaire will be anonymized before being analyzed and your name will never appear in the database.** **Your answers will be used to better understand hepatitis C testing in Pakistan.**

**SECTION A-SOCIODEMOGAPHICS**

A1. Do you consent for your answers to be used in this study?

1. Yes
2. No (*if no stop phone call)*

**SECTION B-SOCIODEMOGAPHICS**

B1. How old are you?

/ / _/ years old

B2. Gender

1. Male

2. Female

3. Other

B3. Which ethnic group do you belong to?

1. Punjabi
2. Pashtun
3. Sindhi
4. Seraiki
5. Muhajir
6. Baloch
7. Other (please indicate) ______

B4. What is the highest level of education that you have completed?

1. None

2. Primary (1- 4 classes)

3. Secondary (school, technical school, vocational school)

4. University or Higher Education

**SECTION C – STUDY TESTING**

C1. Did you complete the hepatitis C testing that was offered to you as part of this study?

1. Yes

2. No

C1a. If yes, what was the result?

1. Positive
2. Negative
3. Invalid/Indeterminate
4. Don’t Know, have forgotten

C1b. If no, why not?

1. Didn’t want to test/wasn’t interested
2. Forgot to get tested
3. Afraid of testing
4. Didn’t have time
5. Other __________
6. Don’t know

C2. In the future, would you test yourself at home if you have a hepatitis C self-testing kit and instructions on how to do it?

1. Yes
2. No
3. Don’t know

**SECTION D – Follow up**

If the participant reports an HCV RDT positive result the AKU staff will arrange a time for the team to come to their house to do the blood draw for RNA and liver staging. For those participants in the control group whom report being RDT+ or RDT- the AKU staff will also confirm these results with the Memon Goth Hospital.

If the participant reports not having taken a test the AKU staff will arrange a time for the team to come to their house to conduct the RDT and further testing.
