# Supplementary material for: Protocol for a cluster randomised controlled trial of secondary distribution of hepatitis C self-testing within the context of a house-to-house hepatitis C micro-elimination programme in Karachi, Pakistan
Source: BMC Public Health. 2022 Apr 9;22:696. doi: 10.1186/s12889-022-13125-9 (PMC8994067; doi:10.1186/s12889-022-13125-9)
Supplement: Supplementary file 2 — Additional file 2. [file 12889_2022_13125_MOESM2_ESM.docx]

## Face to face follow up interview for subset of participants

**HEPATITIS C SELF-TESTING STUDY**

**PERCEPTIONS OF TESTING PARTICIPANT SURVEY**

**STUDY ID:**

**SURVEY DATE:** / ___/ ___/ __/ (day/month/year)

***Conducted at time of follow up to see if participant used self test or went to local site to get tested and their perceptions of the testing process**

**INFORMATION TO PARTICIPANTS**

**This questionnaire will be anonymized before being analyzed and your name will never appear in the database.** **Your answers will be used to better understand hepatitis C testing in Pakistan.**

**SECTION A-SOCIODEMOGAPHICS**

A1. How old are you?

/ / _/ years old

A2. Gender

1. Male

2. Female

3. Other

A3. Which ethnic group do you belong to?

1. Punjabi
2. Pashtun
3. Sindhi
4. Seraiki
5. Muhajir
6. Baloch
7. Other (please indicate) ______

A4. What is the highest level of education that you have completed?

1. None

2. Primary (1- 4 classes)

3. Secondary (school, technical school, vocational school)

4. University or Higher Education

A5. In the last 12 months, have you injected drugs (like heroin, morphine, opium)?

1. Once

2. More than once

3. Never in the last 12 months

4. Decline to answer

A6. In the last 12 months, have you taken any substance by snorting it?

1. Once
2. More than once
3. Never in the last 12 months
4. Decline to answer

A7. Do you have any tattoos?

1. Yes

2. No

3. Decline to answer

A8. Do you go to the barber shop for haircuts/grooming?

1. Yes

2. No

A8a. (*If yes to question A8)* When you are at the barber shop does the barber use a new packaged blade to provide the services for you?

1. Yes

2. No

A8b. (*If yes to question A8)*  When at the barbershop have you ever experienced any injury which caused you to bleed while you were getting your services done?

1. Yes

2. No

**SECTION B – HEPATITIS C TESTING**

B1. Have you ever been tested for hepatitis C before this study?

1. Yes

2. No

3. Don’t know

B1a. If yes, how were you tested?

1. Clinic
2. Hospital
3. Self-test
4. Other _________
5. Don’t know

B1b. If no, why not?

1. Didn’t see myself at risk
2. Didn’t know how to get tested
3. Have not been interested
4. Do not have time to go to a testing centre
5. Afraid of testing hepatitis C positive
6. Afraid of stigma and/or discrimination if I go to a testing centre and ask for a hepatitis C test
7. Other __________
8. Don’t know

B2. Where would you prefer to be tested for hepatitis C?

1. By myself at home
2. At home with someone I trust
3. By myself at a healthcare clinic
4. In a community centre by community-based organization staff
5. In a healthcare clinic by a healthcare worker
6. No preference
7. Prefer not to get tested for hepatitis C
8. Other; specify: ___________

**SECTION D – KNOWLEDGE OF HEPATITIS C**

D1. Hepatitis C is caused by a virus.

1. True
2. False
3. Don’t understand
4. Don’t know

D3. The easiest way to get or give hepatitis C is through deep cuts and sharing needles/syringes.

1. True
2. False
3. Don’t understand
4. Don’t know

D3. Once you are infected with hepatitis C virus, it is possible for you to be a chronic carrier of the disease.

1. True
2. False
3. Don’t understand
4. Don’t know

D4. What can hepatitis C do?

1. Infect cells in the liver
2. Cause inflammation of the liver
3. Cause liver cancer
4. All of the above answers
5. Answers 1 and 2 only
6. Don’t understand
7. Don’t know

D5. Do you know what tests are needed to diagnose hepatitis C before someone can start treatment? Please select all that apply

1. A test to see if you have been exposed to hepatitis C
2. A test to confirm if you have active hepatitis C
3. A test to check on the health of your liver
4. There are no tests needed before you can start treatment
5. No idea

D6. Do you know if people can be treated and cured for hepatitis C?

1. Yes, there is a treatment, but not sure about cure
2. Yes, there is treatment and cure
3. Not sure if treatment or cure
4. There is no treatment or cure
5. No idea

D7. Do you know if there is treatment available for hepatitis C in your community/near your community?

1. Yes
2. Yes, but not nearby
3. No
4. No idea

**SECTION D – STUDY TESTING**

D1. Did you complete the hepatitis C testing that was offered to you as part of this study?

1. Yes

2. No

D1a. If yes, what was the result?

1. Positive
2. Negative
3. Invalid/Indeterminate
4. Don’t Know, have forgotten

D1b. If no, why not?

1. Didn’t want to test/wasn’t interested
2. Forgot to get tested
3. Afraid of testing
4. Didn’t have time
5. Other __________
6. Don’t know

D2a. (*version of question for control group)* Did you ask anyone any question about hepatitis C testing?

1. Yes, via phone
2. Yes, online through searching the internet
3. Yes, person who performed the test
4. Yes, friend or family member
5. Yes; others, specify: ______________
6. No

D2b. (*version of question for intervention group)* Did you ask anyone any question about hepatitis C testing?

1. Yes, via phone
2. Yes, online through searching the internet
3. Yes, friend or family member
4. Yes; others, specify: ______________
5. No

D2. Did you ask anyone any questions about HCV testing?

1. Yes, via phone
2. Yes, to person who delivered or performed the test
3. Yes, friend or family member
4. Yes, other______________
5. No

*D3. (If answered Yes in question D1)* How would you rate the hepatitis C testing you were offered in each of the following categories?

Not very easy Average Very easy

How easy was the testing process? 1 2 3 4 5

Not very convenient Average Very convenient

How convenient was the testing process? 1 2 3 4 5

Not very private Average Very private

How private did you think the testing process was? 1 2 3 4 5

Not very trustworthy Average Very trustworthy

How much do you feel you can trust the test results? 1 2 3 4 5

Not very secure Average Very secure

How secure did you feel during the testing process? 1 2 3 4 5

Not very stressful Average Very stressful

How stressful was the testing process? 1 2 3 4 5

Not very easy Average Very easy

If you needed further care, how easy was it to access it? 1 2 3 4 5 Did not need it

D4. *(If answered Yes in question A1)* Did you feel you could understand the result of your test?

1. Yes
2. No

D4a. *(If answered Yes in question D4, question for intervention arm only)* What do you think have helped you to understand the result of your test (select all that apply)?

1. The explanation from my household member who was explained by AKU how the HCVST works
2. The printed instructions for use that came with the HCV self-test
3. Video instructions on how to perform a self-test
4. Being able to communicate with the AKU study team by phone
5. Other; specify: ____________

D4b. *(If answered No in question D4, question for intervention arm only)* Why do you think you were unable to understand the result of your test? Select all that apply

1. The explanation from my household member who was explained by AKU how the HCVST works was not easy to understand
2. The printed instructions for use that came with the HCV self-test were not easy to understand
3. Video instructions on how to perform a self-test was not easy to understand
4. Communication with the AKU study team by phone were not easy to understand
5. Others; specify: ____________

D5. *(If answered Positive, Invalid or Don’t know in question D1a)* Did you feel you knew what steps you needed to take to be further linked to hepatitis C care after you got the result of your test?

1. Yes
2. No

D6. *(If answered No in question D5)* What do you think would have helped you to know what steps you need to take to be further linked to care?

1. A list of clinics near me that provide HCV care with their contact information
2. More information on how community-based organizations near me could help me navigate how to be linked to care
3. A video explaining how I could get linked to care
4. Information/guidance given to us by the community leaders.
5. Information/guidance given by the local mosque imam
6. Others; specify: _________

D7. In the future, where would you prefer to be tested for hepatitis C?

1. By myself at home
2. At home with someone I trust
3. By myself at a healthcare clinic
4. In a community centre by community-based organization staff
5. In a healthcare clinic by a healthcare worker
6. No preference
7. Prefer not to get tested for hepatitis C
8. Other; specify: ___________

D8. In the future, would you test yourself at home if you have a hepatitis C self-testing kit and instructions on how to do it?

1. Yes
2. No
3. Don’t know

D8a. *(If answered Yes in question D8)* If yes, how often do you think you would test yourself?

1. More than once every 6 months
2. Once every 6 months
3. Once a year
4. Once every 2 years
5. Don’t know
6. Others, specify: _______________
